# Supplementary material for: Suppression of HSF1 activity by wildtype p53 creates a driving force for p53 loss-of-heterozygosity
Source: Nat Commun. 2021 Jun 29;12:4019. doi: 10.1038/s41467-021-24064-1 (PMC8242083; doi:10.1038/s41467-021-24064-1)
Supplement: Supplementary file 1 — Supplementary Information [file 41467_2021_24064_MOESM1_ESM.pdf]

Supplementary information corresponding to:

Suppression of HSF1 activity by wildtype p53 creates a driving force for p53 loss-of-heterozygosity

Tamara Isermann [1], Özge Çiçek Şener [1,#], Adrian Stender [1,#], Luisa Klemke [1], Nadine Winkler [1], Albrecht Neesse [2], Jinyu Li [3], Florian Wegwitz [4], Ute M. Moll [1,3] and Ramona Schulz-Heddergott [1,*]

1) Institute of Molecular Oncology, University Medical Center Göttingen, 37077 Göttingen, Germany
2) Department of Gastroenterology, Gastrointestinal Oncology and Endocrinology, University Medical Center Göttingen, 37077 Göttingen, Germany
3) Department of Pathology, Stony Brook University, Stony Brook NY 11794, USA
4) Department of Gynecology and Obstetrics, University Medical Center Göttingen, 37077 Göttingen, Germany

* Corresponding author: ramona.schulz-heddergott@med.uni-goettingen.de

[#] contributed equally to this work

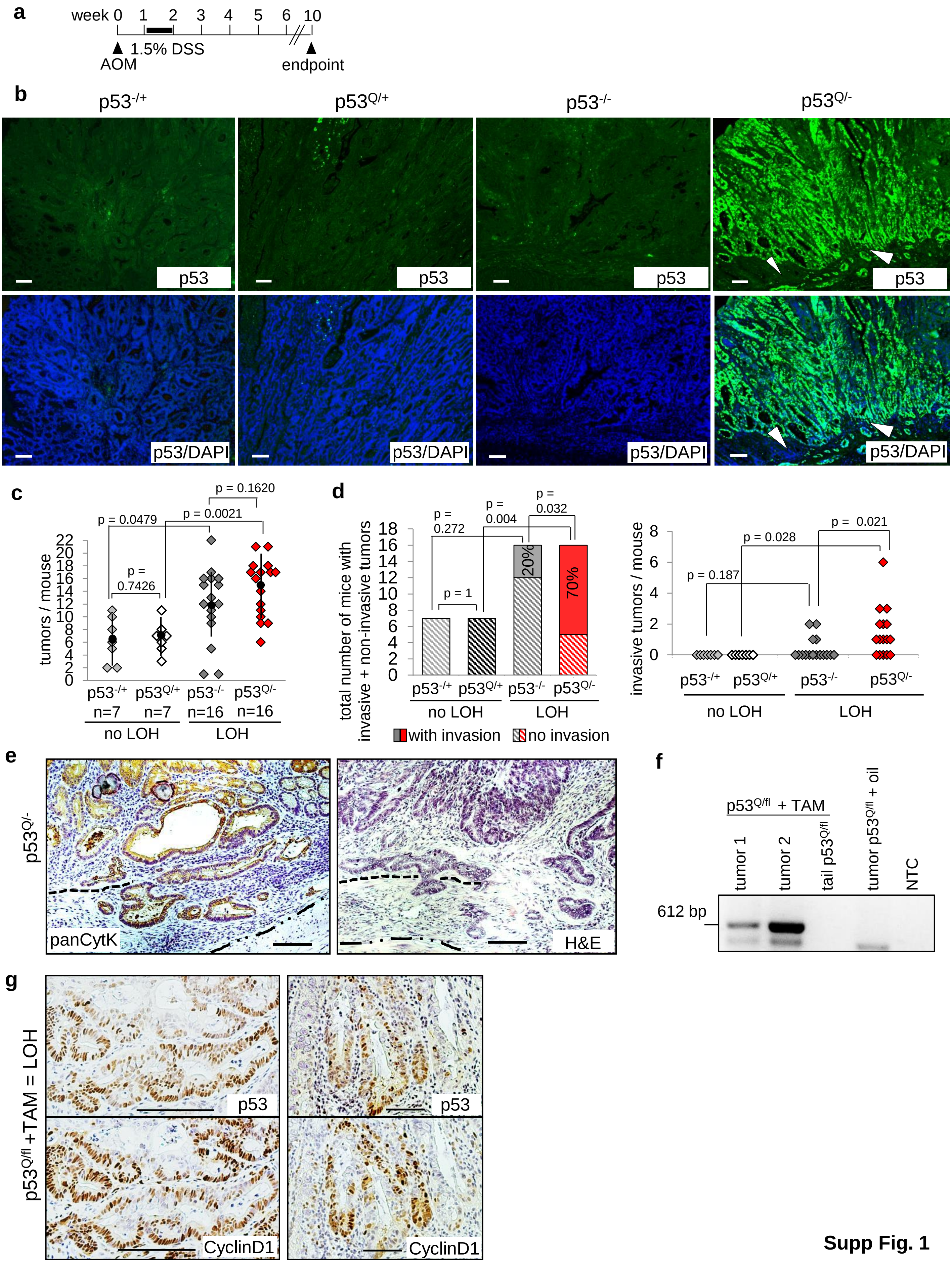
a
week 0 1 2 3 4 5 6 10
1.5% DSS
AOM
endpoint
b
p53-/+
p53Q/+
p53-/-
p53Q/-
p53
p53/DAPI
c
tumors / mouse
p = 0.1620
p = 0.0479
p = 0.0021
p = 0.7426
p53-/+ n=7
p53Q/+ n=7
p53-/- n=16
p53Q/- n=16
no LOH
LOH
d
total number of mice with invasive + non-invasive tumors
p = 0.272
p = 0.004
p = 0.032
p = 1
20%
70%
with invasion
no invasion
invasive tumors / mouse
p = 0.187
p = 0.028
p = 0.021
e
p53Q/-
panCytK
H&E
f
p53Q/fl + TAM
tumor 1
tumor 2
tail p53Q/fl
tumor p53Q/fl + oil
NTC
612 bp
g
p53Q/fl +TAM = LOH
p53
CyclinD1

Supp Fig. 1

Supplementary Figure 1. p53 loss-of-heterozygosity is a prerequisite for mutp53 protein stabilization and enables invasion in colorectal cancer

(a) The humanized GOF *TP53*^{R248Q} allele (p53^{Q}) was paired with the WT p53 allele or the p53null allele[77] in the AOM/DSS colorectal cancer model as we previously described in Schulz-Heddergott et al.[5] to generate heterozygous p53$^{Q/+}$ mice (mimicking 'no LOH') and GOF p53$^{Q/-}$ mice (mimicking p53LOH), with corresponding controls (p53$^{-/+}$ and p53$^{-/-}$ mice). All mice were treated with 1.5% DSS. Time line for p53-proficient (containing one WTp53 allele) and p53-deficient (both p53 alleles are altered) mice used in this study. Endpoint analysis at 10 wks for all mice to avoid losing p53-deficient mice to lymphoma and intestinal obstruction.

(b) Representative immunofluorescence for p53 (green) and DAPI (blue) of CRC tumors from the indicated genotypes at endpoint 10 wks. Occasional p53$^{Q/+}$ tumors show a minor focus of stabilized mutp53, presumably an area that underwent p53LOH. White arrowheads show invasive malignant glands in bowel wall. Scale bars, 100 µm.

(c) Total tumor numbers per mouse of the indicated genotypes at endpoints described in (a). p53$^{-/+}$ and p53$^{Q/+}$ mice (n = 7 each) harbor heterozygous CRC tumors. Tumors from p53$^{-/-}$ and p53$^{Q/-}$ mice (n= 16 each) are homozygous for their TP53 alteration mimicking p53LOH. Dots with vertical black lines represent means ± SD. p values with Student's t-test, two-sided.

(d) (left) Number of mice analyzed for non-invasive and invasive tumors, and (right) invasive tumors per mouse, from the indicated genotypes at endpoint 10 wks.

(left) Calculation of numbers of mice with invasive tumors. A mouse with at least one invasive tumor was counted as positive. p53$^{-/+}$ and p53$^{Q/+}$, n = 7 mice each, p53$^{-/-}$ and p53$^{Q/-}$, n = 16 mice each. p values, Fisher's exact tests for indicated group comparisons. (right) Calculation of number of invasive tumors per mouse. p53$^{-/+}$ from n = total 42 tumors from 7 mice; p53$^{Q/+}$ from n = total 45 tumors from 7 mice; p53$^{-/-}$ from n = total 71 tumors from 16 mice and p53$^{Q/-}$ from n = total 115 tumors from 16 mice. p values, Student's t-test, two-sided.

(e) Representative sections of p53$^{Q/-}$ tumors. panCytK, pan-cytokeratin immunohistochemistry and H&E staining. Dashed line, muscularis mucosae; dashed/dot line, border to muscularis propria. Scale bars, 100 µm.

(f) Detection of the colon-specific recombined *Trp53* allele after TAM-induced excision of exons 2−10 ('Δ' allele, recombined 612 bp fragment). Genomic PCR using a forward primer upstream of the loxP site in intron 1 and a reverse primer downstream of the loxP site in intron10. NTC, non-template control. Source data are provided as Source Data file.

(g) Representative immunohistochemistry of serial sections for p53 and Cyclin D1 from TAM-treated p53$^{Q/fl}$ tumors at 6 wks post-TAM. Tumors show mutp53 stabilization which correlates with Cyclin D1 overexpression. Scale bars, 100 µm.

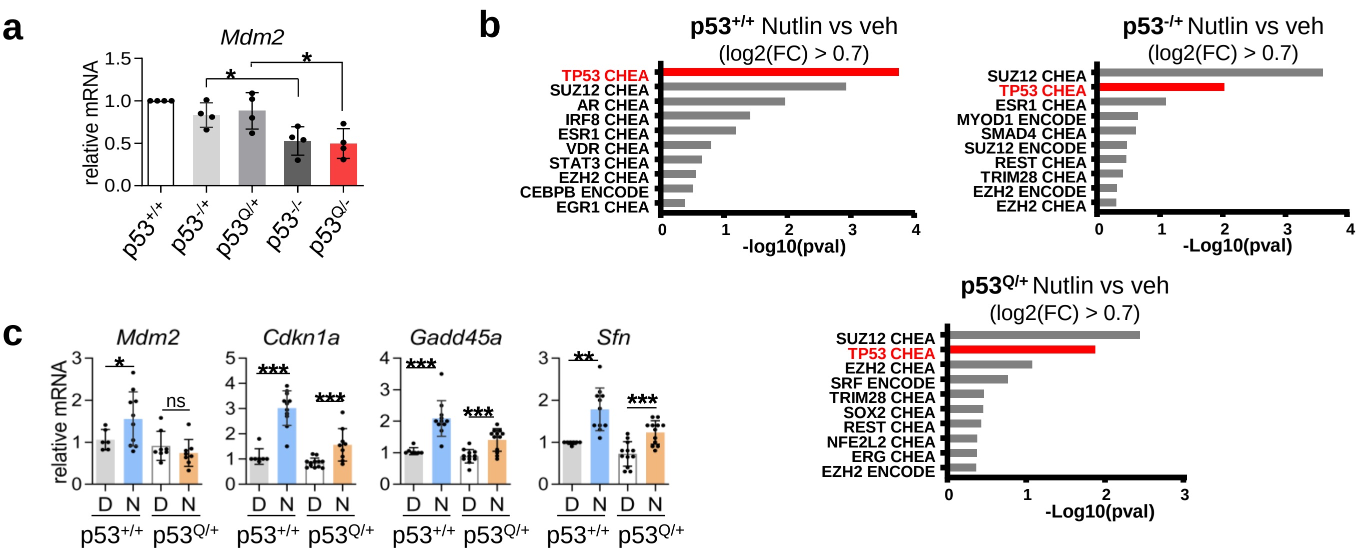

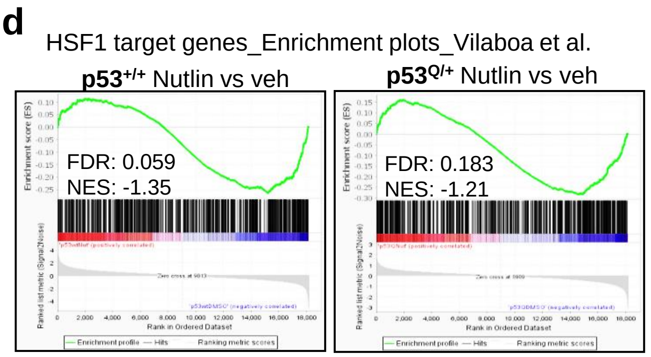

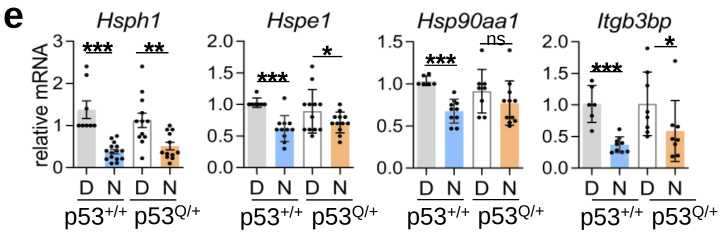

f

GENE_NAME	Log2FC	pVal
Hsph1	-0,8771762	0,01670024
Hspe1	-0,7540928	0,01674211
Hsp90aa1	-0,832613	0,0039347
Hsp90ab1	-0,4405641	0,02203205
Dnaja1	-0,2570268	0,30827905
Stip1	-0,7416906	0,00139609
Itgb3bp	-0,6700182	0,02043334

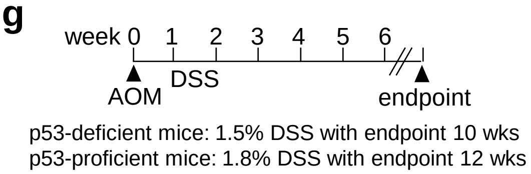

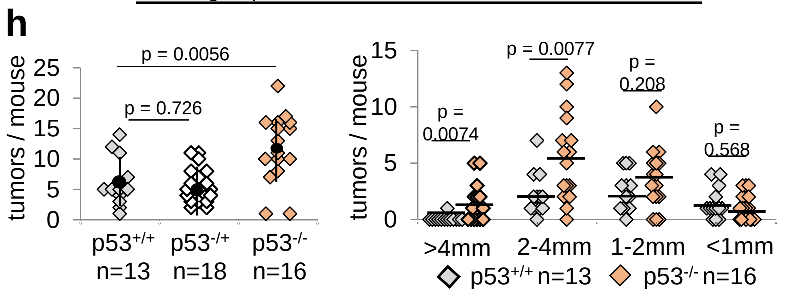

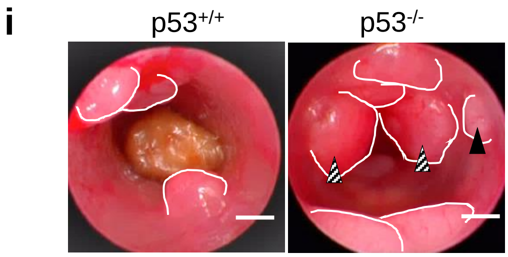

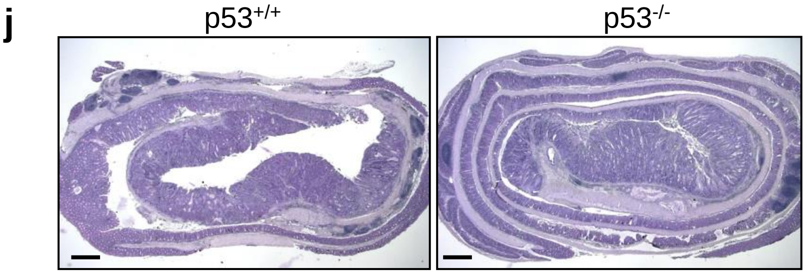

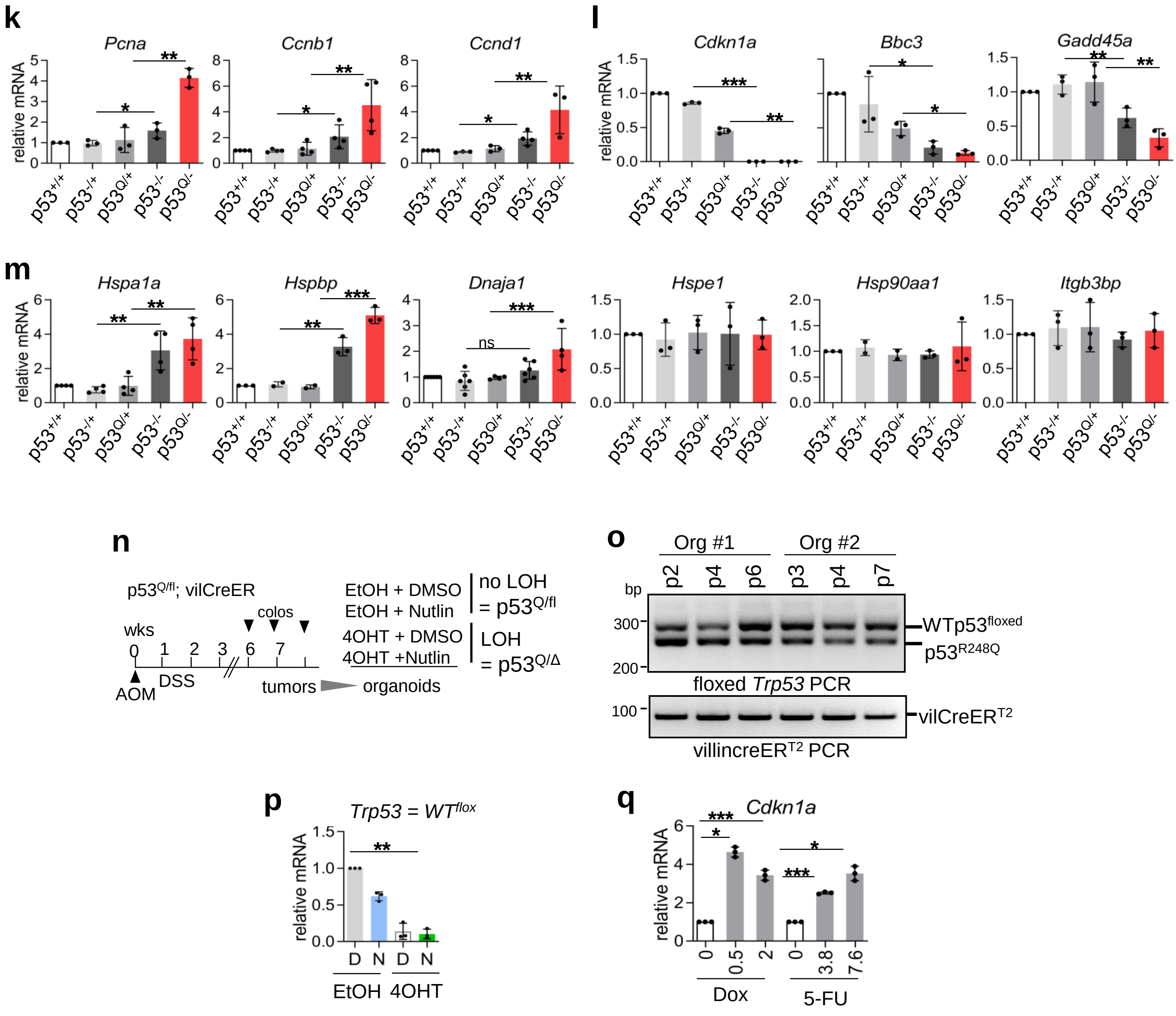
k
Pcna
Ccnb1
Ccnd1
relative mRNA
p53+/+ p53-/+ p53Q/+ p53-/- p53Q/-
l
Cdkn1a
Bbc3
Gadd45a
m
Hspa1a
Hspbp
Dnaja1
Hspe1
Hsp90aa1
Itgb3bp
ns
n
p53Q/fl; vilCreER
wks
colos
AOM
DSS
tumors
organoids
EtOH + DMSO
EtOH + Nutlin
no LOH
= p53Q/fl
4OHT + DMSO
4OHT +Nutlin
LOH
= p53Q/Δ
o
Org #1
Org #2
p2 p4 p6 p3 p4 p7
bp
WTp53floxed
p53R248Q
floxed Trp53 PCR
vilCreERT2
villincreERT2 PCR
p
Trp53 = WTflox
D N D N
EtOH 4OHT
q
Cdkn1a
Dox
5-FU

Supp Fig. 2

Supplementary Figure 2. p53 deletion alone is not sufficient to activate HSF1 *in vivo*

(a) *Mdm2* mRNA levels of untreated CRC tumors (≥ 5 tumors pooled per group). qRT-PCR normalized to *Rplp0* mRNA. Mean ± SD of 3 technical replicates (different cDNA synthesis). Student's t-test, two-sided, p*≤ 0.05.

(b) RNA-seq analysis of +/- Nutlin treated $p53^{+/+}$, $p53^{-/+}$ and $p53^{Q/+}$ tumors from Figure 2b. Upregulated genes (base mean > 10, log2(fold change) > 0.7 and p-value < 0.05) were selected for Enrichr analyses (https://maayanlab.cloud/Enrichr/).

(c, e) mRNA levels of WTp53 target genes (c) and HSF1 target genes (e) of $p53^{Q/+}$ and $p53^{+/+}$ tumors from DMSO (D) and Nutlin (N)-treated mice. Single colonic tumors (n = 3-5 per group) with 2-3 technical replicates for each tumor were used for qRT-PCR, normalized to *Rplp0* mRNA. Mean ± SD.

(d) RNA-seq analysis. Enrichment plots for HSF1 target genes in +/- Nutlin treated $p53^{+/+}$ and $p53^{Q/+}$ tumors. HSF1 target gene list from Vilaboa et al. generated from heat shock-induced Hela cells[64].

(f) Downregulation of individual HSF1 target genes from the RNA-seq analysis in (d).

(g) Scheme of the AOM/DSS colorectal cancer model used in Supplementary Figures 2h-m. Mice of the indicated genotypes were treated with AOM/DSS as outlined. Endpoint analysis at 12 wks for p53-proficient mice to avoid losing them to extraneous reasons such as intestinal obstruction and anal prolapse. Endpoint analysis at 10 wks for p53-deficient mice to avoid losing them to lymphoma.

(h) *left* Total number of colonic tumors per mouse and *right* tumor size distribution of the indicated genotypes. n, total mouse numbers. *left* Dots with vertical black lines represent means ± SD. *right* Horizontal black lines indicate means. Student's t-test, two-sided.

(i) Representative colonoscopy of $p53^{+/+}$ and $p53^{-/-}$ mice at endpoint 10 wks post AOM/DSS. White lines outline tumors. Black arrow indicates an S2 tumor and striped arrows indicate S3 tumors. Tumor scoring was performed according to Becker & Neurath[79]. Scale bars, 1 mm.

(j) Colon sections from 'Swiss roles' of AOM/DSS-treated $p53^{+/+}$ and $p53^{-/-}$ mice. H&E. Scale bars, 400 µM.

(k, l, m) mRNA levels of cell cycle genes (k), wildtype p53 target genes (l) and HSF1 target genes (m) isolated from the indicated genotypes of colonic tumors (pooled samples, n ≥ 5 tumors per genotype). qRT-PCR normalized to *Rplp0* or *Hprt* mRNA. Mean ± SEM of ≥ 3 technical replicates (different cDNA synthesis).

(n) Scheme for treatment of colonic tumor-derived organoids. Heterozygous $p53^{Q/fl}$; $vilCreER^{T2}$ mice were treated with AOM/DSS and tumor burden was visualized via colonoscopy. Tumors arisen between 6-8 wks post AOM were resected and processed for colonic organoid cultures. p53LOH was induced by adding 4OHT (4OH-Tamoxifen) for 24 hrs to activate the $CreER^{T2}$ recombinase and create $p53^{Q/\Delta}$ organoids. EtOH, control treatment (no-LOH, $p53^{Q/fl}$). Two days after p53LOH induction, organoids were treated with 10 µM Nutlin or DMSO for 24 hrs and harvested for analysis.

(o) The heterozygous p53 genotype in CRC organoids is stable. Two randomly chosen organoid cultures (generated from 2 different heterozygous $TP53^{R248Q/fl}$; $vilCreER^{T2}$ mice) were followed during p2-p7 passaging *in vitro*. The p53 floxed allele and the $p53^{Q}$ allele are indicated. Source data are provided as Source Data file.

(p) Incomplete recombination after 4OHT. Residual WT *Trp53* mRNA levels detected in colonic organoids after p53LOH induction by 4OHT treatment. Primers to Exon 1 and Exon 4 specific for the murine WTp53 allele were used. qRT-PCR normalized to *Hprt1* mRNA. Mean ± SD from ≥ 3 independent experiments of ≥ 3 cultures.

(q) *Cdkn1a* mRNA levels of heterozygous (no LOH) $p53^{Q/fl}$; $vilCreER^{T2}$ organoids as in Figure 2f. Organoids were treated as indicated for 24 hrs. Dox=Doxorubucin, 5-FU= Fluorouracil. qRT-PCR normalized to *Hprt* mRNA. Mean ± SD from 2 independent experiments, one includes a technical replicate from the same organoid culture (total n = 3).

(a, c, e, k, l, m, p, q) Relative values are given in [ratio (2^{-ddCT})]. Student's t-test, two-sided, p*≤ 0.05, p**≤ 0.01, p*** ≤0.001; ns, not significant.

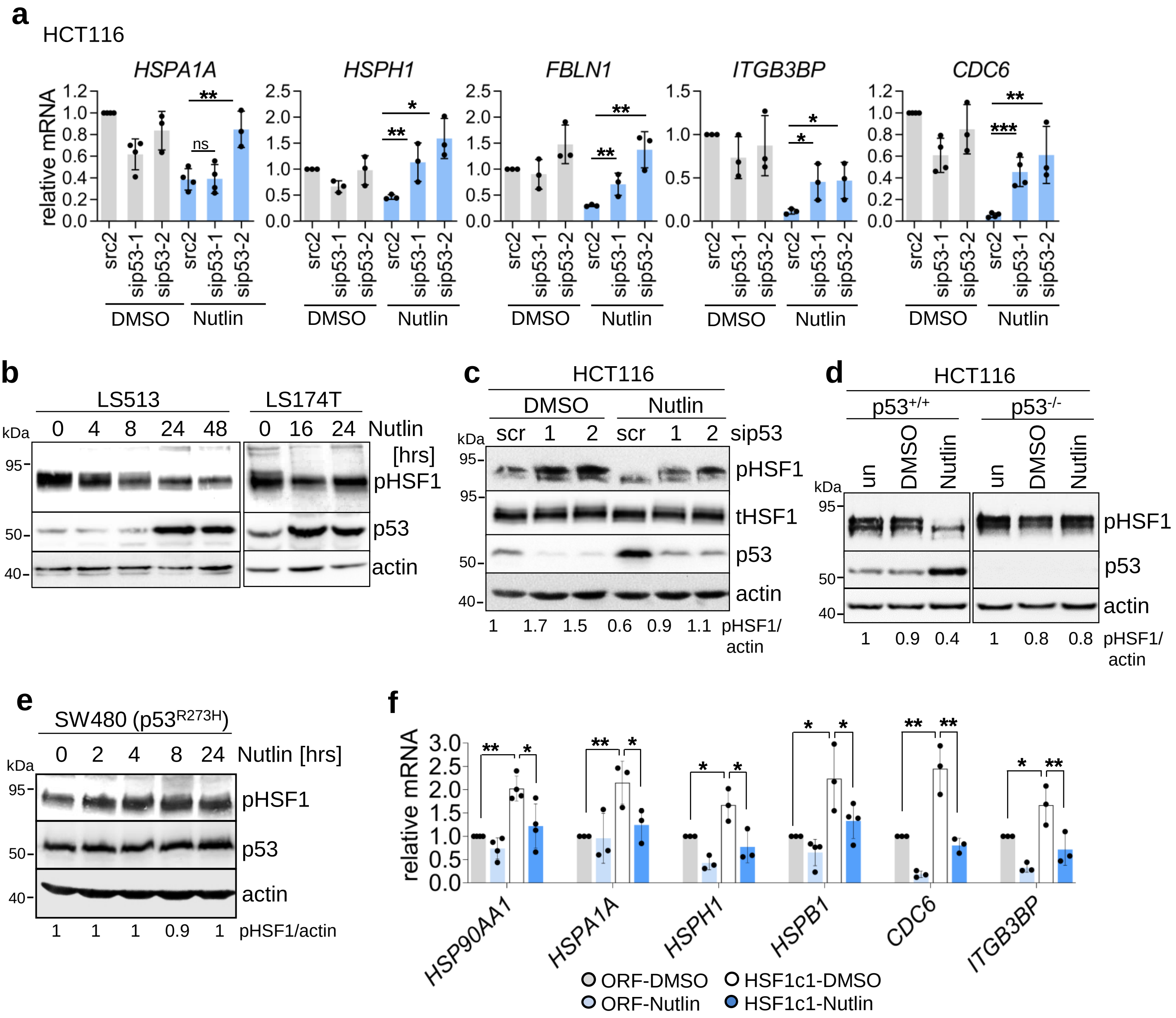
a
HCT116
HSPA1A
HSPH1
FBLN1
ITGB3BP
CDC6
relative mRNA
src2
sip53-1
sip53-2
DMSO
Nutlin
ns
b
LS513
LS174T
kDa
0 4 8 24 48
0 16 24
Nutlin [hrs]
pHSF1
p53
actin
c
HCT116
DMSO
Nutlin
scr 1 2 scr 1 2 sip53
pHSF1
tHSF1
p53
actin
1 1.7 1.5 0.6 0.9 1.1 pHSF1/actin
d
HCT116
p53+/+
p53-/-
un
DMSO
Nutlin
pHSF1
p53
actin
1 0.9 0.4 1 0.8 0.8 pHSF1/actin
e
SW480 (p53R273H)
0 2 4 8 24 Nutlin [hrs]
pHSF1
p53
actin
1 1 1 0.9 1 pHSF1/actin
f
relative mRNA
HSP90AA1
HSPA1A
HSPH1
HSPB1
CDC6
ITGB3BP
ORF-DMSO
ORF-Nutlin
HSF1c1-DMSO
HSF1c1-Nutlin

Supp Fig. 3

Supplementary Figure 3. HSF1 activity is repressed by WTp53 in human colorectal cancer cells

(a) p53-induced HSF1 target gene repression is rescued by WTp53 silencing. WTp53 harboring HCT116 cells were transfected with siRNAs for p53 or scrambled control siRNA (scr2) for 48 hrs. Cells were treated with DMSO or 10 µM Nutlin for 24 hrs. qRT-PCRs for the indicated mRNAs were each normalized to *RPLP0* mRNA. Relative values are given in [ratio (2^{-ddCT})]. Mean ±SD of 2 independent experiments, each repeated in triplicates. Student's t-test, two-sided. p*=0.05, p**=0.01, p***=0.001; ns, not significant.

(b) WTp53 harboring LS513 and LS174T cells were treated with DMSO or 10 µM Nutlin for the indicated times. Representative immunoblot analysis for pSer326-HSF1, the key marker of HSF1 activity.

(c) p53 silencing abrogates HSF1 inactivation upon Nutlin. HCT116 cells were transfected with two different siRNAs against p53 or scrambled control siRNA (scr). 48 hrs post-transfection, cells were treated with DMSO or 10 µM Nutlin for 24 hrs. Cell lysates were immunoblotted for pSer326-HSF1, total HSF1 (tHSF1) and p53.

(d) p53 deletion prevents Nutlin-induced HSF1 inactivation. Isogenic HCT116 cells ($p53^{+/+}$ versus $p53^{-/-}$ harboring a p53 Exon2 deletion) were left untreated (un) or treated with DMSO or 10 µM Nutlin for 24 hrs. Representative immunoblots for pSer326-HSF1 and p53.

(e) mutp53-containing CRC cells fails to reduce pSer326-HSF1 after Nutlin. SW480 cells treated +/- Nutlin (20 µM) for the indicated hours. Representative immunoblot.

(f) Stably HSF1-overexpressing HCT116 subclone HSF1c1 and its empty vector clone (ORF) were treated with DMSO or 10 µM Nutlin for 24 hrs. qRT-PCR analysis of the indicated HSF1 target genes. Mean ± SD of ≥ 2 independent experiments, each with technical replicates. Student's t-test, two-sided. p*≤ 0.05, p**≤ 0.01; ns.

(b-e) Actin, loading control. pHSF1/actin, pHSF1 densitometry normalized to loading control. Source data are provided as Source Data file.

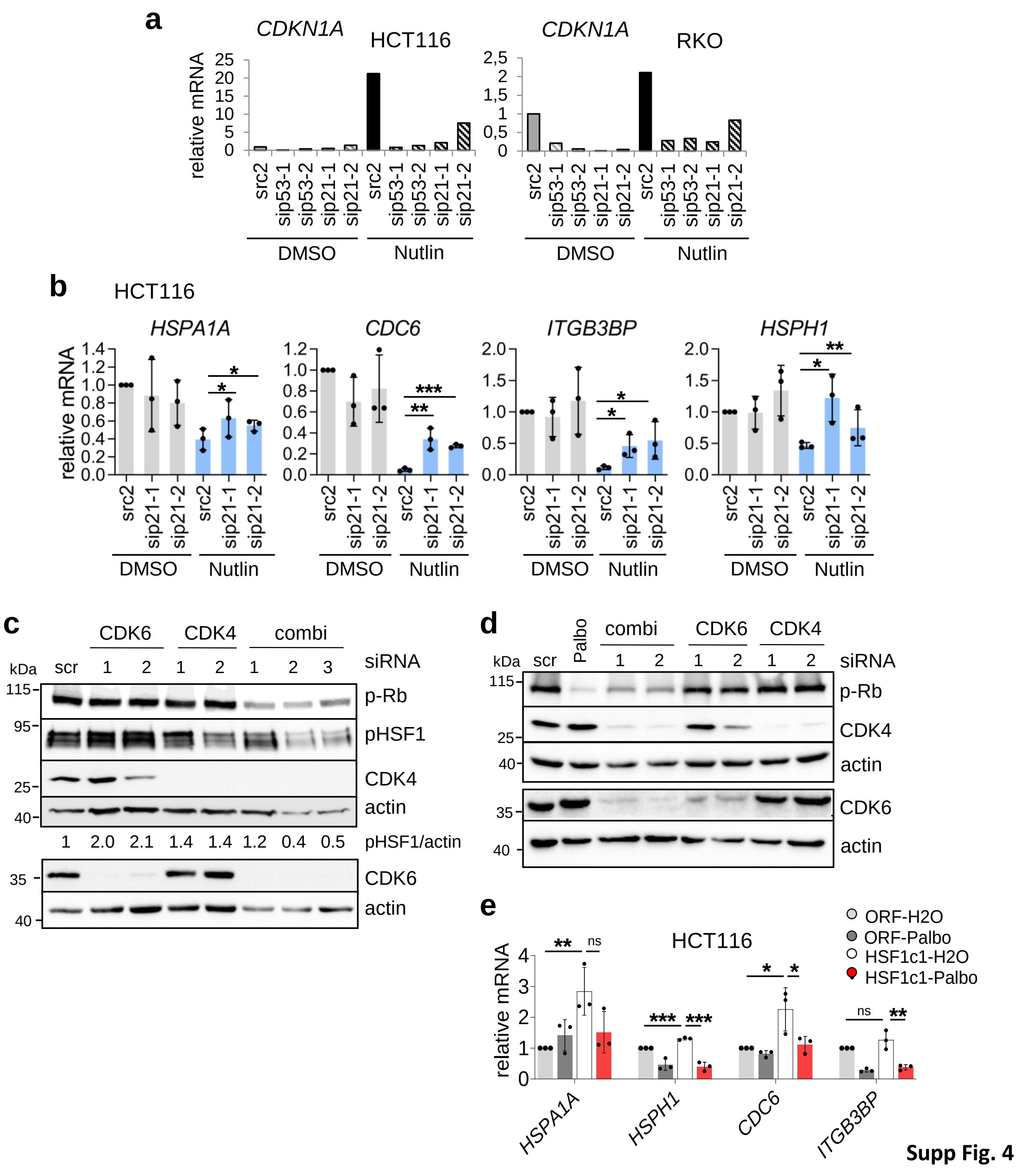

a
CDKN1A
HCT116
CDKN1A
RKO
relative mRNA
src2
sip53-1
sip53-2
sip21-1
sip21-2
DMSO
Nutlin
b
HCT116
HSPA1A
CDC6
ITGB3BP
HSPH1
relative mRNA
DMSO
Nutlin
c
CDK6
CDK4
combi
kDa
scr
siRNA
p-Rb
pHSF1
CDK4
actin
pHSF1/actin
1 2.0 2.1 1.4 1.4 1.2 0.4 0.5
CDK6
actin
d
Palbo
combi
CDK6
CDK4
kDa
scr
siRNA
p-Rb
CDK4
actin
CDK6
actin
e
HCT116
ORF-H2O
ORF-Palbo
HSF1c1-H2O
HSF1c1-Palbo
relative mRNA
HSPA1A
HSPH1
CDC6
ITGB3BP

Supp Fig. 4

Supplementary Figure 4. p53 suppresses HSF1 activity via cyclin-dependent kinase inhibitor *CDKN1A*/p21 in human CRC cells

(a) Analysis of *CDKN1A*/p21 mRNA expression. qRT-PCRs of HCT116 and RKO cells. Cells were transfected with siRNAs against *CDKN1A*/p21 and p53 or scrambled control siRNA (scr2) for 48 hrs, followed by DMSO or 10 µM Nutlin treatment for 24 hrs. Relative values of *CDKN1A* mRNA normalized to *RPLP0* mRNA and relative values given in [ratio (2^{-ddCT})].

(b) Analysis of HSF1 target gene expression in HCT116 cells upon depletion of p21. 48 hrs post- transfection with two siRNAs against *CDKN1A*/p21 or scrambled control siRNA (scr2), HCT116 cells were treated with DMSO or 10 µM Nutlin for 24 hrs. qRT-PCR analysis. Mean ±SD of ≥2 independent experiments, each with technical replicates. Relative values were calculated as in (a).

(c, d) Combined CDK4 and CDK6 silencing lead to HSF1 inactivation. HCT116 cells were transfected with two different siRNAs against CDK4 and/or CDK6 or scrambled control siRNA (scr). 72 hrs post-transfection, cell lysates were immunoblotted as indicated. (D) 10 µM Palbociclib for 24 hrs.

(e) Cell cycle inhibition in HSF1-overexpressing HSF1c1 cells strongly suppress HSF1 target gene expression. HSF1c1 or ORF control clones were exposed to H_2O or Palbociclib (10 µM) for 24 hrs. qRT-PCR analysis of the indicated HSF1 target genes. Mean ± SD of ≥ 2 independent experiments, one with a technical replicate (total n = 3). Relative values given in [ratio (2^{-ddCT})].

(a, b, e) Student's t-test, two-sided. p*≤ 0.05, p**≤ 0.01, p*** ≤0.001; ns, not significant.

(c, d) Actin, loading control. pHSF1/actin, pHSF1 densitometry normalized to loading control. Source data are provided as Source Data file.

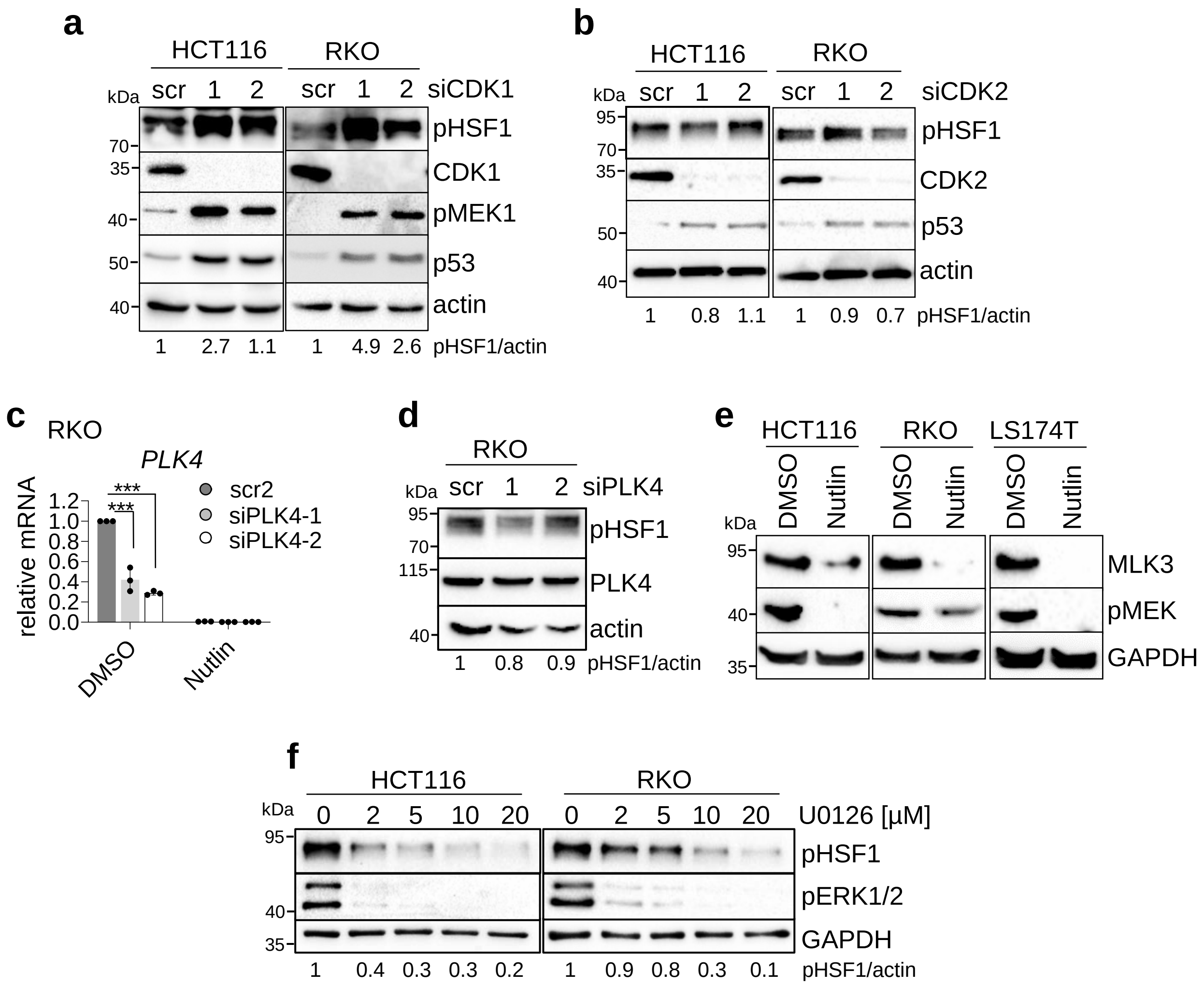
a
HCT116
RKO
kDa
scr 1 2 scr 1 2 siCDK1
pHSF1
CDK1
pMEK1
p53
actin
1 2.7 1.1 1 4.9 2.6 pHSF1/actin
b
HCT116
RKO
kDa
scr 1 2 scr 1 2 siCDK2
pHSF1
CDK2
p53
actin
1 0.8 1.1 1 0.9 0.7 pHSF1/actin
c
RKO
PLK4
relative mRNA
scr2
siPLK4-1
siPLK4-2
***
DMSO
Nutlin
d
RKO
kDa
scr 1 2 siPLK4
pHSF1
PLK4
actin
1 0.8 0.9 pHSF1/actin
e
HCT116
RKO
LS174T
DMSO
Nutlin
kDa
MLK3
pMEK
GAPDH
f
HCT116
RKO
kDa
0 2 5 10 20 0 2 5 10 20 U0126 [µM]
pHSF1
pERK1/2
GAPDH
1 0.4 0.3 0.3 0.2 1 0.9 0.8 0.3 0.1 pHSF1/actin

Supp Fig. 5

Supplementary Figure 5. Cell cycle aberrations activate the MEK pathway and regulate HSF1 activity

(a, b) Depletion of CDK1 (a) and CDK2 (b) fail to abrogate pSer326-HSF1 phosphorylation. The indicated cells were transfected with two different siRNAs each. 72 hrs post-transfection cell lysates were analyzed by immunoblots. Actin, loading control.

(c) *PLK4* silencing in RKO cells. Cells were transfected with two different siRNAs against PLK4 or scrambled control siRNA (scr) for 72 hrs. qRT-PCRs for *PLK4* mRNAs normalized to *RPLP0* mRNA. Relative values given as ratio (2^{-ddCT}). Mean ±SD of 2 independent experiments, one with a technical replicate (total n = 3). Student's t-test, two-sided. $p^* \leq 0.05$, $p^{**} \leq 0.01$, $p^{***} \leq 0.001$.

(d) Despite PLK4 silencing (c), PLK4 protein and pSer326-HSF1 levels are stable, excluding PLK4 as HSF1-activating kinase. Immunoblot analysis of RKO cells from (c).

(e) Activated WTp53 strongly reduces MLK3 protein levels. The indicated CRC cells were treated with DMSO or 10 µM Nutlin for 36 hrs. Immunoblot analysis. Actin, loading control. Source data are provided as Source Data file.

(f) MEK inhibition suppresses HSF1 phosphorylation. HCT116 and RKO cells were treated for 24 hrs with DMSO or U0126 at the indicated concentrations. Immunoblot analysis. pERK1/2, functional control for MEK1 inhibition. GAPDH, loading control.

(a, b, d, f) pHSF1/actin, pHSF1 densitometry normalized to loading control. Source data are provided as Source Data file.

a COADREAD

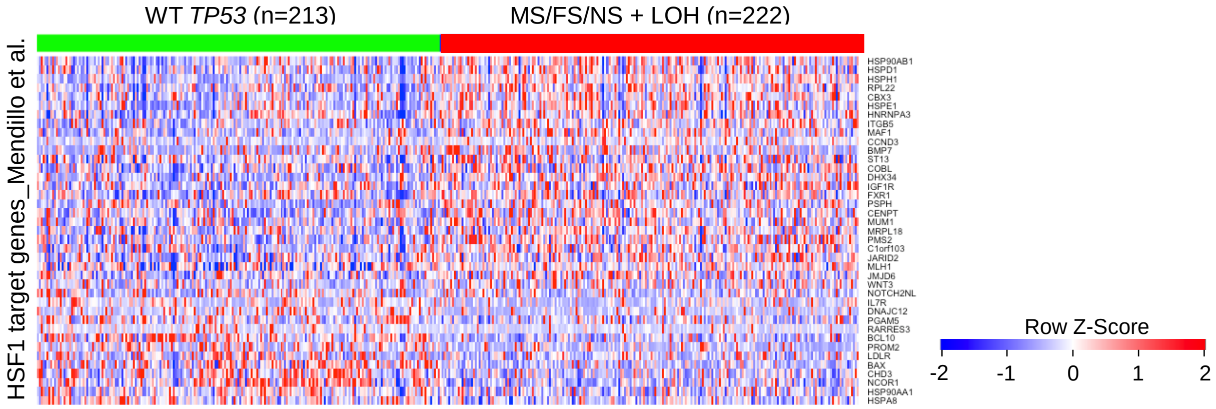

b COADREAD

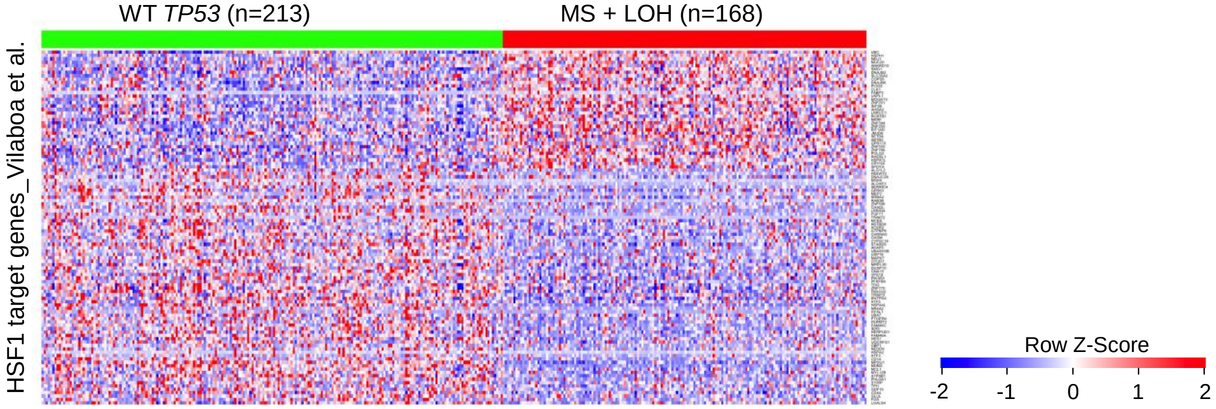

c COADREAD

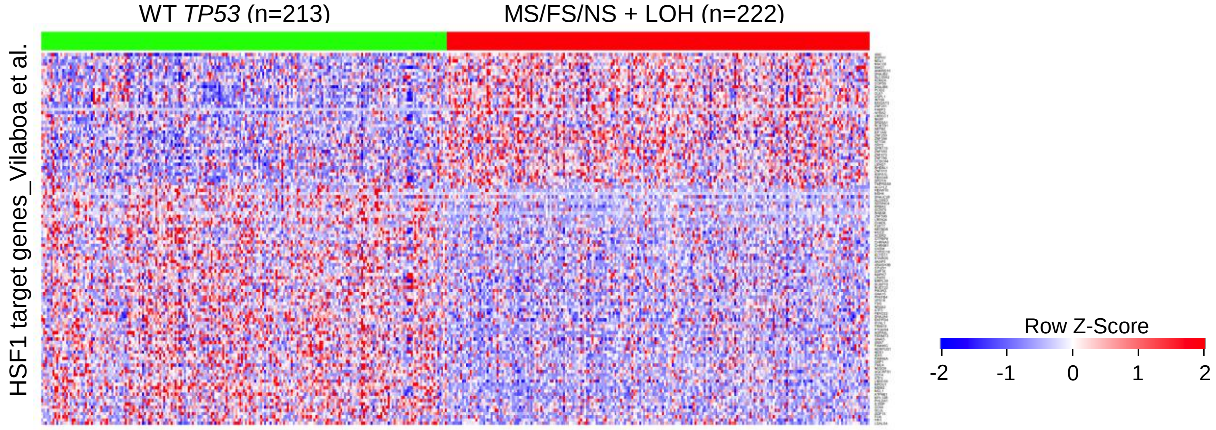

d

n = 433<br>p < .001	WT *TP53*	MS/NS/FS + LOH
N0	147	102
N1A/B/C	37	76
N2A/B	29	42

n = 430<br>p = .0418	WT *TP53*	MS/NS/FS + LOH
M0/X	192	180
M1/1A	19	34
M1B	0	4

e

n = 293<br>p = .0042	WT *TP53* *HSF1* low	MS/NS/FS + LOH *HSF1* high
N0	82	88
N1A/B/C	21	59
N2A/B	17	26

n = 289<br>p = .0018	WT *TP53* *HSF1* low	MS/NS/FS + LOH *HSF1* high
M0/X	114	143
M1/1A	5	26
M1B	0	1

Supp Fig. 6

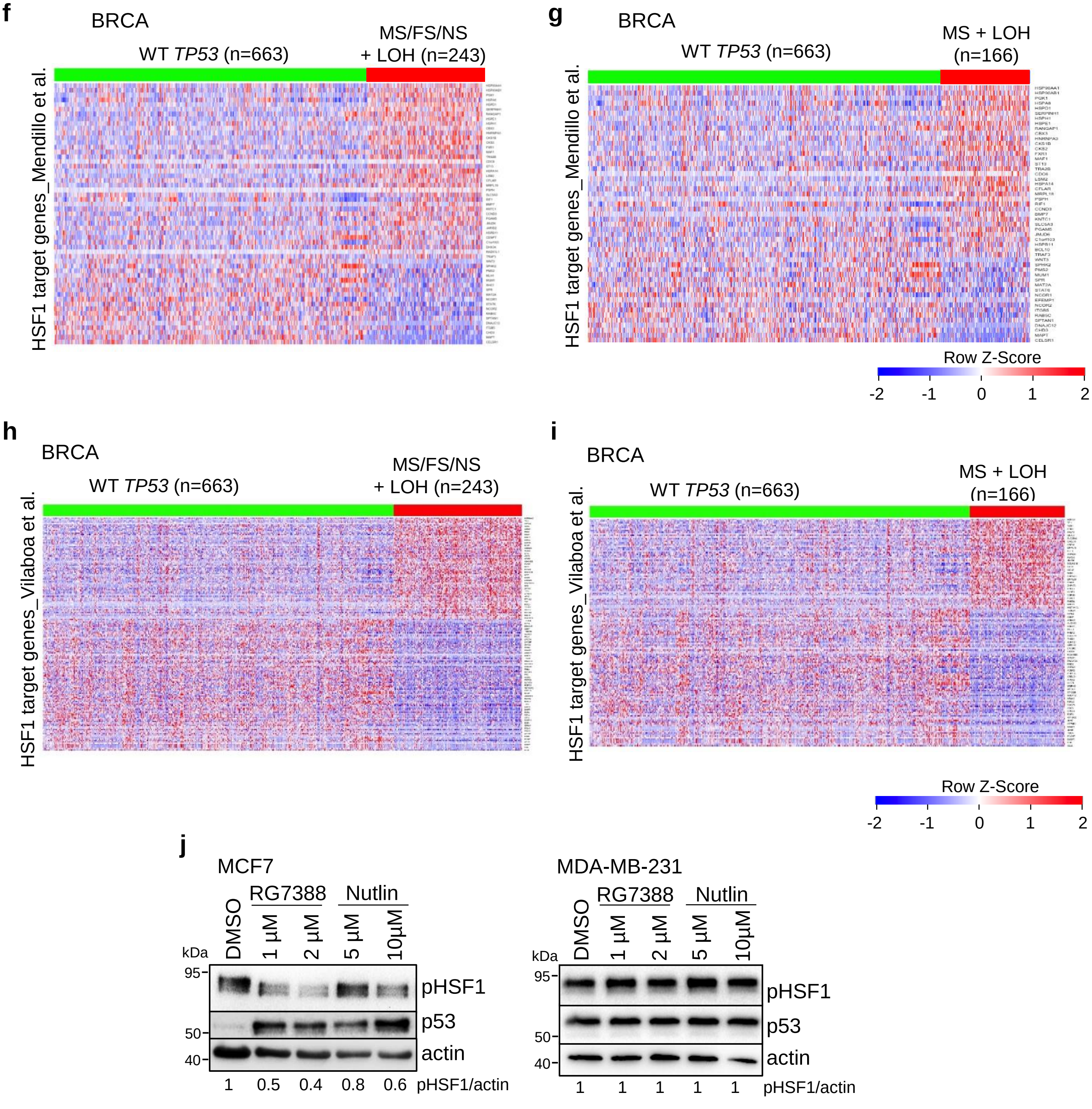
f
BRCA
WT TP53 (n=663)
MS/FS/NS + LOH (n=243)
HSF1 target genes_Mendillo et al.
g
BRCA
WT TP53 (n=663)
MS + LOH (n=166)
HSF1 target genes_Mendillo et al.
Row Z-Score
-2 -1 0 1 2
h
BRCA
WT TP53 (n=663)
MS/FS/NS + LOH (n=243)
HSF1 target genes_Vilaboa et al.
i
BRCA
WT TP53 (n=663)
MS + LOH (n=166)
HSF1 target genes_Vilaboa et al.
Row Z-Score
-2 -1 0 1 2
j
MCF7
RG7388
Nutlin
DMSO
1 µM
2 µM
5 µM
10µM
kDa
95
50
40
pHSF1
p53
actin
1 0.5 0.4 0.8 0.6 pHSF1/actin
MDA-MB-231
RG7388
Nutlin
DMSO
1 µM
2 µM
5 µM
10µM
kDa
95
50
40
pHSF1
p53
actin
1 1 1 1 1 pHSF1/actin

Supp Fig. 6

Supplementary Figure 6. In colorectal and breast cancer patients p53LOH combined with p53 missense mutations upregulates HSF1 activity

(a) Heatmap of HSF1 target genes analyzed from colorectal adenocarcinoma patients (COADREAD cohort, TCGA database). Patients harboring homozygous $TP53^{+/+}$ (WT TP53) were compared to patients harboring all TP53 mutations (MS, missense; FS, frameshift; NS, nonsense) plus p53LOH (shallow deletions) (called MS/FS/NS + LOH). Genes were ordered from top to bottom by their relative Z-scores (upregulation red, downregulation blue) and their log-ranked p-values. A stringently curated HSF1 target gene panel based on HSF1 knockdown and integrated ChiP-seq/RNA-seq criteria from Mendillo et al was used[46].

(b, c) Heatmaps of HSF1 target genes as analyzed in (a). Patients harboring homozygous $TP53^{+/+}$ (WT *TP53*) were compared to patients harboring missense TP53 mutation (MS) plus p53LOH (B) or all TP53 mutations (MS, missense; FS, frameshift; NS, nonsense) plus p53LOH (shallow deletions) (called MS/FS/NS + LOH) (c). Broad HSF1 target gene list from Vilaboa et al was used[64].

(d) Correlation between the p53 mutation status to lymph node stages (N0, N1A/B/C and N2A/B) and metastasis stages (M0/X, M1/1A and M1B) of CRC patients. Patients harboring MS/FS/NS mutations combined with shallow deletions (MS/FS/NS+LOH) were compared to patients with WT TP53. Patient numbers are indicated. Chi-square statistics.

(e) Correlation of CRC patients with WT TP53 and $HSF1^{low}$ or MS/NS/FS+LOH and $HSF1^{high}$ to the N and M stages as in (d). Patient numbers as indicated. Chi-square statistics.

(f, g) Heatmap of HSF1 target genes analyzed from breast cancer patients (BRCA cohort, TCGA database). Patients with homozygous WT TP53 were compared to patients with MS/FS/NS + LOH. Genes were ordered from top to bottom by their relative Z-scores (upregulation red, downregulation blue) and their log-ranked p-values. The HSF1 target gene panel as in (a).

(h, i) Heatmap of HSF1 target genes analyzed from breast cancer patients (BRCA cohort, TCGA database) as analyzed in (f, g). The HSF1 target gene panel as in (b, c).

(j) Breast cancer cells repress pSer326-HSF1 when harboring WTp53 but not mutp53. MCF7 cells harboring homozygous WTp53 and MDA-MB-231 cells harboring homozygous mutp53 R280K were treated with DMSO, Nutlin or Idasanutlin (RG7388) for 24 hrs as indicated. Representative immunoblot analysis. Actin, loading control. pHSF1/actin, pHSF1 densitometry normalized to loading control. Source data are provided as Source Data file.

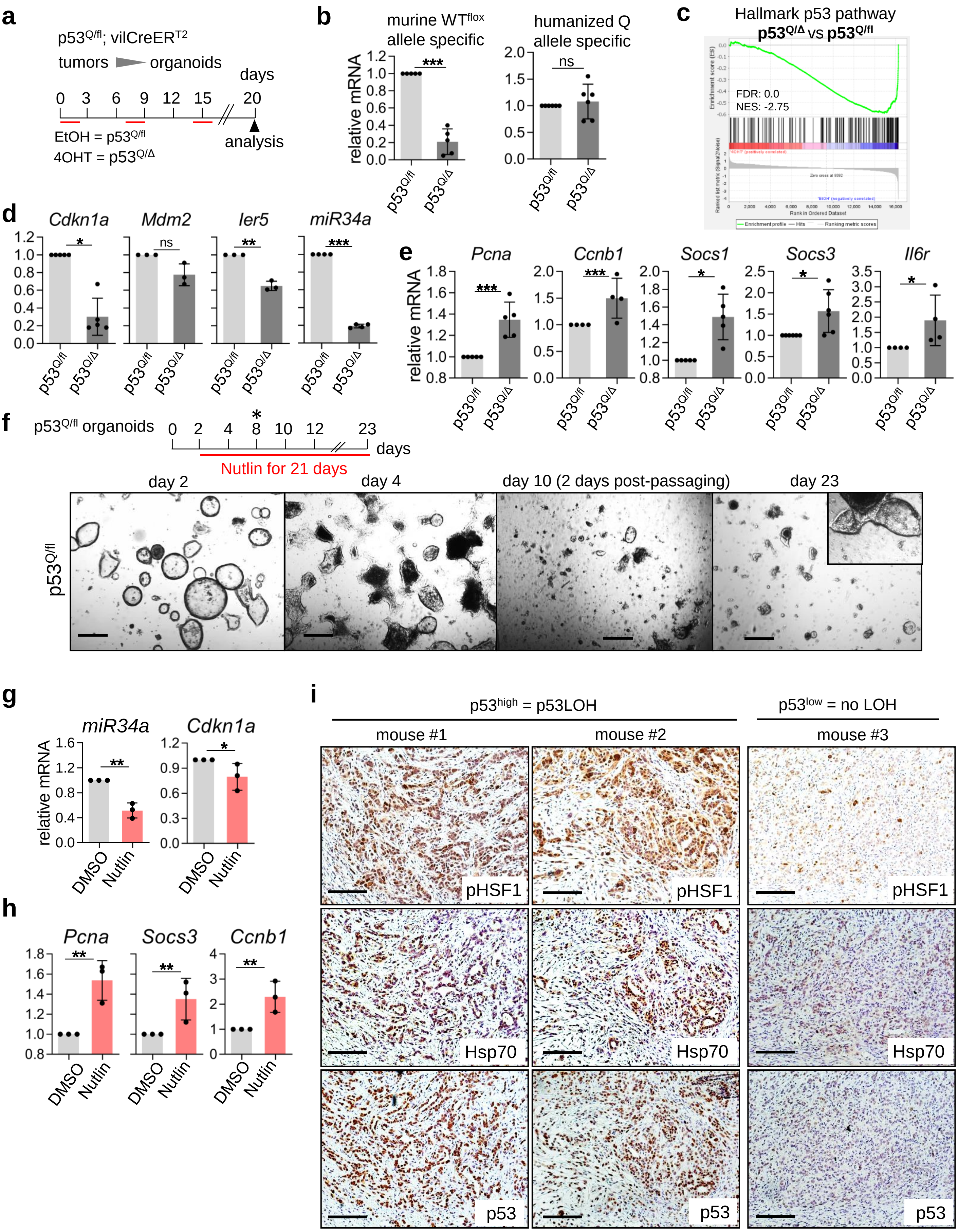
a
p53Q/fl; vilCreERT2
tumors organoids
days
0 3 6 9 12 15 20
EtOH = p53Q/fl
4OHT = p53Q/Δ
analysis
b
murine WTflox allele specific
humanized Q allele specific
relative mRNA
***
ns
p53Q/fl
p53Q/Δ
c
Hallmark p53 pathway
p53Q/Δ vs p53Q/fl
FDR: 0.0
NES: -2.75
d
Cdkn1a
Mdm2
Ier5
miR34a
e
Pcna
Ccnb1
Socs1
Socs3
Il6r
relative mRNA
f
p53Q/fl organoids
0 2 4 8 10 12 23
days
Nutlin for 21 days
day 2
day 4
day 10 (2 days post-passaging)
day 23
p53Q/fl
g
miR34a
Cdkn1a
relative mRNA
DMSO
Nutlin
h
Pcna
Socs3
Ccnb1
i
p53high = p53LOH
p53low = no LOH
mouse #1
mouse #2
mouse #3
pHSF1
Hsp70
p53

Supp Fig. 7

Supplementary Figure 7. Analysis of heterozygous CRC organoids

(a) Treatment scheme used in (b-d) for ‘long-term p53LOH’ ($p53^{Q/\Delta}$) of $p53^{Q/fl}$; vilCreER^{T2} organoids over 20 days, induced by adding weekly 4OHT for 48 hrs (red lines). EtOH, control (no LOH, $p53^{Q/fl}$).

(b) Validation of induced p53LOH after 20 days and 3 weekly pulses of 4OHT. mRNA levels of the murine wildtype *Trp53* allele and the humanized *TP53* Q allele of heterozygous $p53^{Q/fl}$;vilCreER^{T2} organoids treated as in (a). qRT-PCR using species-specific primers, normalized to *Hprt1* mRNA.

(c) RNA-seq analysis of organoids subjected to long-term p53LOH generated in (a). GSEA enrichment plot for the hallmark p53 gene set sorted by NES.

(d, e) mRNA levels of the indicated p53 target genes (d) and proliferative Stat3 target genes (e) in heterozygous $p53^{Q/fl}$;vilCreER^{T2} organoids treated as in (a). qRT-PCR normalized to *Hprt1* or *Rplp0* mRNA.

(f) Long-term chronic Nutlin treatment of heterozygous $p53^{Q/fl}$; vilCreER^{T2} organoids provides selection pressure to induce spontaneous p53LOH. Two days after plating, organoids were treated with 5µM Nutlin/DMSO for 21 days. At day 8 dead organoids induced by Nutlin-activated WTp53 (marked as black structures with complete loss of integrity of the outer epithelial barrier) were removed by splitting the cultures (passaging). Subsequently many organoids, presumably those that had undergone p53LOH, were able to regrow and show invasive morphologic structures with branchings and protrusions. Representative images of different times are shown. Scale bars, 200 µM.

(g, h) Long-term chronic Nutlin treatment to mimic chronic stress for spontaneous p53LOH. mRNA levels of p53 targets (g) and proliferative Stat3 target genes (h) from heterozygous $p53^{Q/fl}$;vilCreER^{T2} organoids. qRT-PCR normalized to *Hprt1* mRNA. Mean ± SD from 2 independent experiments, one includes a technical replicate from the same organoid culture (total n = 3).

(i) Serial sections of three representative pancreatic KPC tumors immunostained for p53, pHSF1 and Hsp70. Scale bars, 100 mm.

(b, d, e) Mean ± SD from ≥ 3 independent experiments of ≥ 3 cultures.

(b, d, e, g, h) Student’s t-test, two-sided. p*≤ 0.05, p**≤ 0.01, p***≤ 0.001; ns, not significant.

Supplementary Table 1, related to online Methods:: Reagents and Resources

REAGENT or RESOURCE	SOURCE	IDENTIFIER
**Antibodies**		
Rabbit polyclonal anti-p53 (FL-393)	Santa Cruz	Cat# sc-6243; RRID:AB_653753
Goat polyclonal anti-αSMA	Abcam	Cat# ab21027, RRID:AB_1951138
Mouse monoclonal anti-E-Cadherin	BD Biosciences	Cat# 610181; RRID:AB_397580
Mouse monoclonal anti-p53 (DO-1)	Santa Cruz	Cat# sc-126; RRID:AB_628082
Rabbit monoclonal phospho-Ser326-HSF1	Abcam	Cat# ab76076; RRID:AB_1310328
Rabbit monoclonal phospho-Ser326-HSF1 (for murine IHC)	Bioss	Cat# bsm-52166R
Rabbit polyclonal anti-HSF1 (H-311)	Santa Cruz	Cat# sc-9144; RRID:AB_2120276
Rabbit monoclonal anti-HSP27 (E1J4D)	Cell Signaling	Cat# 50353; RRID:AB_2799374
Rabbit polyclonal anti-Heat Shock Protein 90alpha	Millipore	Cat# 07-2174; RRID:AB_10807022
Rabbit polyclonal anti-Hsp70	Cell Signaling	Cat# 4872; RRID:AB_2279841
Rabbit polyclonal anti-AKT	Cell Signaling	Cat# 9272; RRID:AB_329827
Mouse monoclonal anti-beta-actin	Abcam	Cat# ab6276; RRID:AB_2223210
Rabbit polyclonal anti-c-Raf	Cell Signaling	Cat# 9422; RRID:AB_390808
Rabbit monoclonal anti-p21 Waf1/Cip1 (12D1)	Cell Signaling	Cat# 2947; RRID:AB_823586
Rabbit monoclonal anti-phospho-Rb (Ser807/811) (D20B12) XP	Cell Signaling	Cat# 8516; RRID: AB_11178658
Rabbit polyclonal anti-phospho-Ser235/236-S6 ribosomal protein	Cell Signaling	Cat# 2211; RRID:AB_331679
Rabbit monoclonal anti-MLK3 [EP1460Y]	Abcam	Cat# ab51068; RRID:AB_881140
Rabbit polyclonal phospho-p-MEK-1/2 (Ser 218/Ser 222)	Santa Cruz	Cat# sc-7995; RRID:AB_2234805
Rabbit monoclonal anti-phospho-p44/42 MAPK (Erk1/2) (Thr202/Tyr204) [D13.14.4E]	Cell Signaling	Cat# 4370, RRID:AB_2315112
Mouse monoclonal anti-GAPDH	Abcam	Cat# ab8245; RRID:AB_2107448

Mouse monoclonal anti-Cdc2 p34	Santa Cruz	Cat# sc-54; RRID:AB_627224
Rabbit monoclonal anti-CDK2	Abcam	Cat# ab32147; RRID:AB_726775
Rabbit monoclonal anti-CDK4	Abcam	Cat# ab68266; RRID:AB_11155340
Rabbit monoclonal anti-CDK6	Cell Signaling	Cat# 3136; RRID:AB_2229289
Rabbit monoclonal anti-CyclinD1	Abcam	Cat# ab134175; RRID:AB_2750906
Rabbit polyclonal anti-PLK4	Protein Technologies	Cat# 12952-1-AP; RRID:AB_2284150
Alexa Fluor®488 Goat anti-rabbit IgG (H+L)	ThermoFisher	Cat# A-11034; RRID:AB_2576217
Alexa Fluor®488 Donkey anti-mouse IgG (H+L)	ThermoFisher	Cat# A-21202; RRID:AB_141607
Alexa Fluor®546 Donkey anti-rabbit IgG (H+L)	ThermoFisher	Cat# A-10040; RRID:AB_2534016
ImmPRESS™ Peroxidase polymer reagent	VectorLabs	Cat# MP-7401, RRID:AB_2336529
**Bacterial and Virus Strains**		
Bacteria: ElectroMAX DH10B cells	Invitrogen/Thermo Fisher Sci.	Cat# 18290-015
**Chemicals, Peptides, and Recombinant Proteins**		
AOM (Azoxymethane)	Sigma Aldrich	Cat# A5486
DSS (Dextran sodium sulfate)	MP Biomedicals	Cat# 160110
TAM (Tamoxifen)	Sigma Aldrich	Cat# T5648
(Z)-4-Hydroxytamoxifen (4-OHT)	Sigma Aldrich	Cat# H7904
Lipofectamine2000	Invitrogen	Cat# 11668-019
Trizol	Invitrogen	Cat# 15596026
Phusion® High-Fidelity DNA Polymerase	Thermo Fisher Sci.	Cat# F530
U0126	Cell Signaling	Cat# 9903S
PD 0332991 isethionate (Palbociclib)	Sigma Aldrich	Cat# PZ0199
RO-3306	Sigma Aldrich	Cat# SML0569
Roscovitine	Cell Signaling	Cat# 9885
RG-7112	SelleckChem	Cat# S7030

Idasanutlin (RG-7388)	SelleckChem	Cat# S7205
Nutlin-3a (MDM2 inhibitor)	BOC Sciences	Cat# 675576-98-4
5-Fluorouracil	Sigma	Cat# F6627
Doxorubicin	Santa Cruz	Cat# sc-200923
Passive Lysis Buffer, 5X	Promega	Cat# E194A
**Experimental Models: Cell Lines**		
HCT116	ATCC	Cat# ATCC® CCL-247™
HCT116-ORF	this work	N/A
HCT116-HSF1c1	this work	N/A
HCT116-HSF1c2	this work	N/A
RKO	ATCC	Cat# ATCC ® CRL-2577™
LS513	ATCC	Cat# ATCC ® CRL-2134™
HCT116 p53-/-	Bunz et al., 1998.	B. Vogelstein, Baltimore
HCT116 p53+/+	Bunz et al., 1998.	B. Vogelstein, Baltimore
LS174T	DSMZ	Cat# ACC 759
SW480	DSMZ	Cat# ACC 313
MCF-7	DSMZ	Cat# ACC 115
MDA-MB-231	DSMZ	Cat# ACC 732
HEK 293 Cell Line human (for viral transfection)	DSMZ	Cat# 85120602
**Experimental Models: Organisms/Strains**		
Mouse: $p53^{LoxP}$ ($p53^{fl}$)	Jonkers J et al., 2001	Jax strain# 008462
Mouse: p53null (-/-) (B6.129S2-Trp53<tm1Tyj>/J)	Jacks T et al., 1994 or The Jackson Laboratory	Jax strain# 002101
Mouse: $p53^{R248Q}$	Hanel et al., 2013	N/A
Mouse: $p53^{floxR248Q}$ ($p53^{floxQ}$)	Alexandrova et al., 2015	N/A
Mouse: villin:$CreER^{T2}$	N/A	Jax strain# 020282
Mouse: C57BL/6NJ	N/A	Jax strain# 005304

KPC mouse	Hasselluhn et al. 2020	PMID: 31878349
**Oligonucleotides**		
Primers for QPCR and genotyping, see below	this paper	Table S1.
siRNA MLK3 Silencer® Select	Ambion	Pool of IDs: 8814+8815+8816
siRNA CDC2 Silencer® Select	Ambion	ID: 464
siRNA CDC2 Silencer® Select	Ambion	ID: 465
siRNA CDK2 Silencer® Select	Ambion	ID: 205
siRNA CDK2 Silencer® Select	Ambion	ID: 206
siRNA CDK4 Silencer® Select	Ambion	ID: s2824
siRNA CDK4 Silencer® Select	Ambion	ID: s2822
siRNA CDK6 Silencer® Select	Ambion	ID: s51
siRNA CDK6 Silencer® Select	Ambion	ID: s53
siRNA PLK4 Silencer® Select	Ambion	ID: 21083
siRNA PLK4 Silencer® Select	Ambion	ID: 21084
siRNA TP53 Silencer® Select	Ambion	ID: s605
siRNA TP53 Silencer® Select	Ambion	ID: s607
siRNA CDKN1A Silencer® Select	Ambion	ID: 415
siRNA CDKN1A Silencer® Select	Ambion	ID: 417
siRNA Negative Control No. 2 (src2) Silencer® Select siRNA	Ambion	Cat# 4390847
**Recombinant DNA**		
pSUPER control vector for shRNA	OligoEngine	Cat# VEC-PBS-0002
pSUPER-p53 for shp53	OligoEngine	Cat# VEC-P53-0001
pMD2.G	Addgene	Plasmid #12259
pCMV-R8.91	PlasmidFactory Bielefeld	Kramer et al., 2017. PMID: 27834954
Precision LentiORF positive control	Dharmacon	

Precision LentiORF HSF1 w/o Stop Codon, Lentiviral	Dharmacon	Catalog# OHS5898-202620209; Clone Id:PLOHS_100008319
pGL4.41[*luc2P/HSE/Hygro*] vector	Promega	Cat# E3751
pRL Renilla Luciferase Control Reporter Vectors	Promega	Cat# E2241
Software and Algorithms		
ImageJ software	Open source	https://imagej.net/Welcome<br>PMID 22930834
GraphPadPRISM®	Graphpad Software, Inc.	https://www.graphpad.com/
Image Lab™ Software	Biorad	http://www.bio-rad.com/de-de/product/image-lab-software

Supplementary Table 2, related to online Methods: Primers for qPCR and genotyping.

Gene	Origin	Forward	Reverse
**qPCR**			
HSP90AA1	Human	5'-GCCCAGAGTGCTGAATACCC	5'-GTGGAAGGGCTGTTTCCAGA
HSPA1A	Human	5'-TCAAGGGCAAGATCAGCGAG	5'-TGATGGGGTTACACACCTGC
HSPH1	Human	5'-ACTGCTTGTTCAAGAGGGCTGTGA	5'-AACATCCACACCCACACACATGCT
HSPB1	Human	5'-GGAGTGGTCGCAGTGGTTAG	5'-ATGTAGCCATGCTCGTCCTG
CDC6	Human	5'-TAAAAGCCCTGCCTCTCAGC	5'-TGAGTGAGGGGGACCATTCT
ITGB3BP	Human	5'-TCCCGAATCTCAGAATGCCTG	5'-TGACAAGTTCCAGTTGTTGGAG
RBBP5	Human	5'-AACTCAGCCAGCCCTTGAC	5'-GGCCACATGATGGCAAAGTG
BST2	Human	5'-AGGAGCTTGAGGGAGAGATCA	5'- AGGACGGACCTTCCAAGATG
RPLP0 (36B4)	Human	5'-GATTGGCTACCCAACTGTTG	5'-CAGGGGCAGCAGCCACAAA
TP53	Human	5'-AAGTCTAGAGCCACCGTCCA	5'-CAGTCTGGCTGCCAATCCA
FBNL1	Human	5'-CCGCAACTGCCAAGACATTGAT	5'-GACCGTGTCTGTCTTCTCCTG
CDKN1A	Human	5'-TAGGCGGTTGAATGAGAGG	5'-AAGTGGGGAGGAGGAAGTAG
CDK1	Human	5'-TTTTCAGAGCTTTGGGCACT	5'-CCATTTTGCCAGAAATTCGT
CDK2	Human	5'-GGATGCCTCTGCTCTCACTG	5'-ACAGGGTCACCACCTCATGG
CDC25c	Human	5'-GTATCTGGGAGGACACATCCAGGG	5'-CAAGTTGGTAGCCTGTTGGTTTG
PLK4	Human	5'-CAAGCGGCGGGAGATTTTCA	5'-CAGCTCTGTAGACACCAGCAA
MLK3	Human	5'-CACACCCCCAGCACTCAAT	5'-CGTCTTGAGCGAGAAGCAGA
STIP1	Human	5'-CCGACCTTCATCAAGGGTTATAC	5'-GGTTGTACTGCGCCATCATA
DNAJA1	Human	5'-ACCCAAATGAAGGAGAGAAGGTAAA	5'-GTACCTCGGCAATTGGGACA
DNAJB1	Human	5'-TCGGACGAGGAGATCAAGCG	5'-AACATGGCATGAGGGTCTCC
HSF1	Human	5'-AAGGAGGTGCTGCCCAAGTA	5'-ACTCCGTGTCGTCTCTCTCT
Tp53 (Ex4-Ex5)	Human	5'-GTTTCCGTCTGGGCTTCTT	5'-GTGCTGTGACTGCTTGTAGAT
Trp53 (Ex1-Ex3/4)	Mouse	5'-GTGCTCACCCTGGCTAAAGT	5'-CAGTGAGGTGATGGCAGGAT
Ccnd1	Mouse	5'-GGAGCTGCTGCAAATGGAAC	5'-CAGTCCGGGTCACACTTGA
Ccnb1	Mouse	5'-CAGGGTCGTGAAGTGACTGG	5'-GGCACACAACTGTTCTGCAT
Mdm2	Mouse	5'-TAG CAG CCA AGA AAG CGT GA	5'-ATG AGG TGT CCA GTC TTG CC
Pcna	Mouse	5'-AGTGGAGAGCTTGGCAATGG	5'-TCAGGTACCTCAGAGCAAACG
Cdkn1a (p21)	Mouse	5'-GTGGCCTTGTCGCTGTCTT	5'- GCGCTTGGAGTGATAGAAATCTG
Gadd45a	Mouse	5'-GGCGTGTACGAGGCTGCCAA	5'-TGTCGTTCTCGCAGCAGAACG
Bbc3	Mouse	5'-TTCTCCGGAGTGTTCATGCC	5'-ATACAGCGGAGGGCATCAGG
Puma	Mouse	5'-CCTGTCACCAGCCCAGCAGC	5'-CCCTCCAGGGTGAGGGTC
Sfn	Mouse	5'-GCCCGGTCAGCCTACCAGGA	5'-CGGCTGTCCACAGCGTCAGG
MLK3	Mouse	5'-TCCGCCTCTCACAAACAACA	5'-ATACGGCTACGGAGTGGTGA

IER5	Mouse	5'-CACCGACGAGGAGATGGAGA	5'-AGTCCCGAGAAGCTAGACCC
Zmat3	Mouse	5'-AGAGTCACTCATTCTCGGAC	5'-GAACTCCACCTCTTCGCCAG
Pai1	Mouse	5'-ACTTCACAAGTCTTTCCGACCAAG	5'-GGCCCATGAAGAGGATTGTC
HspH1	Mouse	5'-AGACCATCGCCAACGAGTTC	5'-ACATGACCTTTATTCCCACGC
HspE	Mouse	5'-GGAGTGCTGCCGAAACTGTA	5'- CCAACTTTCACACTGACAGGC
Hsp90AA1	Mouse	5'-CGTCTCGTGCGTGTTCATTC	5'-CCAGAGCGTCCGATGAATTG
Hsp90AB1	Mouse	5'-TCTAATGCTTCAGATGCCCTGG	5'-CGTGCCAGACTTAGCAATGG
HspA1A	Mouse	5'- GATTTGTTTTGCAGGACAGC	5'-GGGGAGAGTCCAAACACAAA
HspA2	Mouse	5'-TCAAGCGCCTCACCCAACTA	5'-GGAGACATCCTGACTGGTCG
DNAJA1	Mouse	5'-AGGGTCATGGAGAACGCATC	5'-GGCTCCAGTCCTGGTTCTTG
STIP1	Mouse	5'-CCCAAGCCAGAACCAATGGA	5'-TCTCAAAGTGCACAGCTGCTT
SOCS1	Mouse	5'-CGAGTAGGATGGTAGCACGC	5'-AAGGTGCGGAAGTGAGTGTC
SOCS3	Mouse	5'-TGTCGGAAGACTGTCAACGG	5'-AGGAAGAAGCCAATCTGCCC
Itgb3bp	Mouse	5'-GTATACAGGCTTTGGAGGGCA	5'-TGACAGTTGTCAGACTTGAAGGT
pre-miR34a	Mouse	5'-GGTAGGGTCCACTACACATCTTTC	5'-CTAGGGCAGTATACTTGCTGATTG
Snai1	Mouse	5'-CTTGTGTCTGCACGACCTG	5'-GGTTGGAGCGGTCAGCAAA
Vim	Mouse	5'-GGATCAGCTCACCAACGACA	5'-AAGGTCAAGACGTGCCAGAG
Zeb1	Mouse	5'-TTCTCTCCACTGTGAATCCGT	5'-TTGCTCACCTGCCCGTATTG
Snai2	Mouse	5'-GGCTGCTTCAAGGACACATT	5'-GTGCCCTCAGGTTTGATCTG
Hprt1	Mouse	5'-GCTTCCTCCTCAGACCGCTT	5'-CCAGCAGGTCAGCAAAGAACT
RplpO (36B4)	Mouse	5'-GCAGATCGGGTACCCAACTGTT	5'-CAGCAGCCGCAAATGCAGATG
**genotyping**			
Trp53^{R248Q} = WT	Mouse	5'-GGAAGTCCTTTGCCCTGAA	5'-CACTGAAAAAGACCTGGCAACC
TP53^{R248Q} = humanized Q	Hu/Mus	5'-AAGGGTGCAGTTATGCCTCA (Human)	~
Trp53 (X6-X7) = WT	Mouse	5'-AGCGTGGTGGTACCTTATGAGC	5'-GGATGGTGGTATACTCAGAGCC
Trp53 (neo-X7) = Del	Mouse	5'-GCTATCAGGACATAGCGTTGGC	~
villinCreER^{T2} = transgene	Mouse	5'-CAA GCC TGG CTC GAC GGC C	5´- CGC GAA CAT CTT CAG GTT CT
Trp53^{flox} = WT and floxed site in intron 1 of Trp53<br><br>(F1 - R1)	Mouse	5'- GGT TAA ACC CAG CTT GAC CA<br><br>(F1)	5- GGA GGC AGA GAC AGT TGG AG (R1)
Trp53^{flox} = recombined<br><br>(F1 and R10)	Mouse	5'- GGT TAA ACC CAG CTT GAC CA<br><br>(F1)	5- GAA GAC AGA AAA GGG GAG GG (R10)
