# Supplementary material for: Suppression of HSF1 activity by wildtype p53 creates a driving force for p53 loss-of-heterozygosity
Source: Nat Commun. 2021 Jun 29;12:4019. doi: 10.1038/s41467-021-24064-1 (PMC8242083; doi:10.1038/s41467-021-24064-1)
Supplement: Supplementary file 3 — Description of Additional Supplementary Files [file 41467_2021_24064_MOESM3_ESM.pdf]

Description of Additional Supplementary Files

File Name: Supplementary Data 1
Description: HSF1 UP gene list_after HS_HeLa
