# Supplementary material for: Suppression of HSF1 activity by wildtype p53 creates a driving force for p53 loss-of-heterozygosity
Source: Nat Commun. 2021 Jun 29;12:4019. doi: 10.1038/s41467-021-24064-1 (PMC8242083; doi:10.1038/s41467-021-24064-1)
Supplement: Supplementary file 5 — Reporting Summary [file 41467_2021_24064_MOESM5_ESM.pdf]

nature research

Corresponding author(s): Ramona Schulz-Heddergott

Last updated by author(s): May 14th 2021

Reporting Summary

Nature Research wishes to improve the reproducibility of the work that we publish. This form provides structure for consistency and transparency in reporting. For further information on Nature Research policies, see our Editorial Policies and the Editorial Policy Checklist.

Statistics

For all statistical analyses, confirm that the following items are present in the figure legend, table legend, main text, or Methods section.

n/a	Confirmed	
☐	☒	The exact sample size (*n*) for each experimental group/condition, given as a discrete number and unit of measurement
☐	☒	A statement on whether measurements were taken from distinct samples or whether the same sample was measured repeatedly
☐	☒	The statistical test(s) used AND whether they are one- or two-sided<br>*Only common tests should be described solely by name; describe more complex techniques in the Methods section.*
☒	☐	A description of all covariates tested
☒	☐	A description of any assumptions or corrections, such as tests of normality and adjustment for multiple comparisons
☐	☒	A full description of the statistical parameters including central tendency (e.g. means) or other basic estimates (e.g. regression coefficient) AND variation (e.g. standard deviation) or associated estimates of uncertainty (e.g. confidence intervals)
☐	☒	For null hypothesis testing, the test statistic (e.g. *F*, *t*, *r*) with confidence intervals, effect sizes, degrees of freedom and *P* value noted<br>*Give P values as exact values whenever suitable.*
☒	☐	For Bayesian analysis, information on the choice of priors and Markov chain Monte Carlo settings
☐	☒	For hierarchical and complex designs, identification of the appropriate level for tests and full reporting of outcomes
☒	☐	Estimates of effect sizes (e.g. Cohen's *d*, Pearson's *r*), indicating how they were calculated

Our web collection on statistics for biologists contains articles on many of the points above.

Software and code

Policy information about availability of computer code

Data collection	No software was used.
Data analysis	Image Lab™ (BioRad, version 5.2.1), ZENblue software V3.0 Zeiss, GraphPadPrism (version 5.04), Adobe Photoshop (version 12.0 x32), RNA STAR, version 2.5.2b-2

For manuscripts utilizing custom algorithms or software that are central to the research but not yet described in published literature, software must be made available to editors and reviewers. We strongly encourage code deposition in a community repository (e.g. GitHub). See the Nature Research guidelines for submitting code & software for further information.

Data

Policy information about availability of data

All manuscripts must include a data availability statement. This statement should provide the following information, where applicable:

- Accession codes, unique identifiers, or web links for publicly available datasets
- A list of figures that have associated raw data
- A description of any restrictions on data availability

We used TCGA (The Cancer Genome Atlas) colorectal cancer (COARDREAD) and breast cancer (BRCA) databases in this analysis. Human genomic data including RNA expression, DNA copy number alteration, gene mutation, and clinical information was downloaded from cBioPortal (http://www.cbioportal.org/study/summary?id=coadread_tcga_pan_can_atlas_2018). Study names: Colorectal adenocarcinoma (TCGA, PanCancer Atlas, 594 total samples) and Breast Invasive Carcinoma (TCGA, PanCancer Atlas, 1084 total samples).
mRNAseq data of murine Nutlin-treated tumors were deposited in the public repository Annotare Array Express database (https://www.ebi.ac.uk/fg/annotare/login/) under the accession number E-MTAB-10041. mRNAseq data of murine organoids after p53LOH generated during this study are available under the accession number E-MTAB-10416 in the public repository Annotare Array Express database (https://www.ebi.ac.uk/fg/annotare/login/).

Source data with full scan images of all immunoblots and DNA gels are provided as source data file.
Authors can confirm that all relevant data are included in the paper and/or in its supplementary information files. All remaining data are available from the authors upon request.

Field-specific reporting

Please select the one below that is the best fit for your research. If you are not sure, read the appropriate sections before making your selection.

☒ Life sciences ☐ Behavioural & social sciences ☐ Ecological, evolutionary & environmental sciences

For a reference copy of the document with all sections, see nature.com/documents/nr-reporting-summary-flat.pdf

Life sciences study design

All studies must disclose on these points even when the disclosure is negative.

Sample size	Sample size of mice was determined by power analysis to achieve a minimum effect size of 0.5 with a P value of less than 0.05 and all sample sizes were appropriate for assumption of normal distribution.<br>Sample size of patient data analysis was predicted by the availability in the database.
Data exclusions	No data were excluded.
Replication	Replication information are provided in the corresponding Figure legend. For Figures with representative images, information are provided in the reproducibility section which indicates how many times each experiment was repeated independently with similar results. Similar results were mainly obtained from at least three independent experiments. All attempts at replication were successful.
Randomization	All experimental animals were allocated into experimental groups randomly. A clinical trial is not applied in this study.
Blinding	For experiments with imaging and microscopy, manual counting was done with Image J, samples were blinded and labeled with numbers for the analyzing investigator. Samples for expression analysis and immunoblots were not blinded.

Reporting for specific materials, systems and methods

We require information from authors about some types of materials, experimental systems and methods used in many studies. Here, indicate whether each material, system or method listed is relevant to your study. If you are not sure if a list item applies to your research, read the appropriate section before selecting a response.

Materials & experimental systems

n/a	Involved in the study	
☐	☒	Antibodies
☐	☒	Eukaryotic cell lines
☒	☐	Palaeontology and archaeology
☐	☒	Animals and other organisms
☒	☐	Human research participants
☒	☐	Clinical data
☒	☐	Dual use research of concern

Methods

n/a	Involved in the study	
☒	☐	ChIP-seq
☒	☐	Flow cytometry
☒	☐	MRI-based neuroimaging

Antibodies

Antibodies used

Rabbit polyclonal anti-p53 (FL-393) , Santa Cruz, Cat# sc-6243; RRID:AB_653753, 1:400 for IHC/IF, 1:1000 for WB;
Goat polyclonal anti-αSMA , Abcam, Cat# ab21027, RRID:AB_1951138, 1:600 for IHC/IF;
Mouse monoclonal anti-E-Cadherin, BD Biosciences, Cat# 610181; RRID:AB_397580, 1:500 for IF;
Mouse monoclonal anti-p53 (DO-1), Santa Cruz, Cat# sc-126; RRID:AB_628082, 1:1000 for WB;
Rabbit monoclonal phospho-Ser326-HSF1, Abcam, Cat# ab76076; RRID:AB_1310328, 1:1000 for WB;
Rabbit monoclonal phospho-Ser326-HSF1, Bioss, Cat# bsm-52166R, 1:2000 for IHC;
Rabbit polyclonal anti-HSF1 (H-311), Santa Cruz, Cat# sc-9144; RRID:AB_2120276, 1:1000 for WB;
Rabbit monoclonal anti-HSP27 (E1J4D), Cell Signaling, Cat# 50353; RRID:AB_2799374, 1:2000 for WB;
Rabbit polyclonal anti-Heat Shock Protein 90alpha, Millipore, Cat# 07-2174; RRID:AB_10807022, 1:1000 for WB;
Rabbit polyclonal anti-Hsp70, Cell Signaling, Cat# 4872; RRID:AB_2279841, 1:1000 for WB;
Rabbit polyclonal anti-AKT Cell Signaling, Cat# 9272; RRID:AB_329827, 1:1000 for WB;
Mouse monoclonal anti-beta-actin, Abcam, Cat# ab6276; RRID:AB_2223210, 1:2000 for WB;
Rabbit polyclonal anti-c-Raf, Cell Signaling, Cat# 9422; RRID:AB_390808, 1:1000 for WB;
Rabbit monoclonal anti-p21 Waf1/Cip1 (12D1),Cell Signaling, Cat# 2947; RRID:AB_823586, 1:500 for WB;

Rabbit monoclonal anti-phospho-Rb (Ser807/811) (D20B12) XP, Cell Signaling, Cat# 8516; RRID: AB_11178658, 1:2000 for WB, 1:500 for IHC;
Rabbit polyclonal anti-phospho-Ser235/236-S6 ribosomal protein, Cell Signaling, Cat# 2211; RRID:AB_331679, 1:1000 for WB;
Rabbit monoclonal anti-MLK3 [EP1460Y], Abcam, Cat# ab51068; RRID:AB_881140, 1:500 for WB;
Rabbit polyclonal phospho-p-MEK-1/2 (Ser 218/Ser 222), Santa Cruz, Cat# sc-7995; RRID:AB_2234805, 1:1000 for WB;
Rabbit monoclonal anti-phospho-p44/42 MAPK (Erk1/2) (Thr202/Tyr204) [D13.14.4E], Cell Signaling, Cat# 4370, RRID:AB_2315112, 1:1000 for WB;
Mouse monoclonal anti-GAPDH, Abcam, Cat# ab8245; RRID:AB_2107448, 1:500 for WB;
Mouse monoclonal anti-Cdc2 p34, Santa Cruz, Cat# sc-54; RRID:AB_627224, 1:500 for WB;
Rabbit monoclonal anti-CDK2, Abcam, Cat# ab32147; RRID:AB_726775, 1:2000 for WB;
Rabbit monoclonal anti-CDK4, Abcam, Cat# ab68266; RRID:AB_11155340, 1:1000 for WB;
Rabbit monoclonal anti-CDK6, Cell Signaling, Cat# 3136; RRID:AB_2229289, 1:500 for WB;
Rabbit monoclonal anti-CyclinD1, Abcam, Cat# ab134175; RRID:AB_2750906, 1:2000 for WB;
Rabbit polyclonal anti-PLK4, Protein Technologies, Cat# 12952-1-AP; RRID:AB_2284150, 1:1000 for WB;
Alexa Fluor®488 Goat anti-rabbit IgG (H+L), ThermoFisher, Cat# A-11034; RRID:AB_2576217, 1:600 for IF
Alexa Fluor®488 Donkey anti-mouse IgG (H+L), ThermoFisher, Cat# A-21202; RRID:AB_141607, 1:600 for IF;
Alexa Fluor®546 Donkey anti-rabbit IgG (H+L), ThermoFisher, Cat# A-10040; RRID:AB_2534016, 1:600 for IF;
Alexa Fluor®647, Donkey anti-Rabbit IgG (H+L), ThermoFisher, Cat# A-31573; 1:600 for IF;
ImmPRESS™ Peroxidase polymer reagent, VectorLabs, Cat# MP-7401, RRID:AB_2336529, 1:1000 for IHC;

Validation

All antibodies were purchased from companies, and the corresponding blot sample figures, concentrations and conditions were available on the manufacturer's website. For key antibodies (such as pHSF1-Ser326) additional validations were performed in our laboratory before using cells silenced against HSF1 with siRNA.

Eukaryotic cell lines

Policy information about cell lines

Cell line source(s)

HCT116, ATCC, Cat# ATCC® CCL-247™;
HCT116-ORF, this work;
HCT116-HSF1c1, this work;
HCT116-HSF1c2, this work;
RKO, ATCC, Cat# ATCC ® CRL-2577™;
LS513, ATCC, Cat# ATCC ® CRL-2134™;
HCT116 p53-/-, Bunz et al., 1998;
HCT116 p53+/+, Bunz et al., 1998;
LS174T, DSMZ, Cat# ACC 759;
SW480, DSMZ, Cat# ACC 313;
MCF-7 DSMZ, Cat# ACC 115;
MDA-MB-231, Cat# ACC 732;
HEK 293 Cell Line human (for viral transfection), DSMZ, Cat# 85120602

Authentication

Cell line authentication was performed by the German Cell line collection (DSMZ) using DNA profiling using 17 different and highly polymorphic STR (Short Tandem Repeat) loci. In addition, they have tested our human samples for the presence of mitochondrial DNA sequences from rodent cells such as mouse, rat, Chinese and Syrian hamster. Animal cell line samples have been subjected to the procedure of Cytochrome C Subunit I (COI) DNA Barcoding for identification of the species. All cell lines used in this study showed a full-matching STR profile of the corresponding cell line in the reference database, authentic*.

Mycoplasma contamination

All cell lines used are tested regularly every 3 month and were tested as negative.

Commonly misidentified lines
(See ICLAC register)

not used.

Animals and other organisms

Policy information about studies involving animals; ARRIVE guidelines recommended for reporting animal research

Laboratory animals

Mice were kept under pathogen-free barrier temperature-controlled (20 - 22°C) conditions, with a 12 hrs day and 12 hrs dark/light cycle, with free access to water and standard rodent diet.
All mouse strains were maintained on a C57BL/6 background for at least 6 generations. For experiments, randomly assigned 10 wk old males and females weighing at least 20 g were used.
Mouse: p53LoxP (p53fl), Jonkers J et al., 2001 , Jax strain# 008462;
Mouse: p53null (-/-) (B6.129S2-Trp53<tm1Tyj>/J), Jacks T et al., 1994 or The Jackson Laboratory, Jax strain# 002101;
Mouse: p53R248Q, Hanel et al., 2013
Mouse: p53floxR248Q (p53floxQ), Alexandrova et al., 2015,
Mouse: villin:CreERT2, Jax strain# 020282
Mouse: C57BL/6NJ, Jax strain# 005304

Wild animals

Not used.

Field-collected samples	Not done.
Ethics oversight	Experiments using animal materials were approved by institutional (Göttingen University Medical Center Ethikkommission) and state (Niedersächsisches Landesamt für Verbraucherschutz und Lebensmittelsicherheit, LAVES, Lower Saxony, Germany) committees, ensuring that all experiments conform to the relevant regulatory standards and protocols.

Note that full information on the approval of the study protocol must also be provided in the manuscript.
